# Protein disulfide isomerases are promising targets for predicting the survival and tumor progression in glioma patients

**DOI:** 10.18632/aging.102748

**Published:** 2020-02-05

**Authors:** Zhigang Peng, Yu Chen, Hui Cao, Hecun Zou, Xin Wan, Wenjing Zeng, Yanling Liu, Jiaqing Hu, Nan Zhang, Zhiwei Xia, Zhixiong Liu, Quan Cheng

**Affiliations:** 1 Department of Neurosurgery, Xiangya Hospital, Central South University, Changsha 410008, Hunan, P. R. China; 2 Department of Neurosurgery, The First Affiliated Hospital of Zhengzhou University, Zhengzhou 450001, Henan, P. R. China; 3 Department of Psychiatry, The Second People's Hospital of Hunan Province, The Affiliated Hospital of Hunan University of Chinese Medicine, Changsha 410007, Hunan, P. R. China; 4 Department of Clinical Pharmacology, Xiangya Hospital, Central South University, Changsha 410008, Hunan, P. R. China; 5 Department of Emergency, The Second People’s Hospital of Hunan Province, The Affiliated Hospital of Hunan University of Chinese Medicine, Changsha 410007, Hunan, P. R. China; 6 School of Bioinformatics Science and Technology, Harbin Medical University, Harbin 150081, Heilongjiang, P. R. China; 7 Department of Neurology, Xiangya Hospital, Central South University, Changsha 410008, Hunan, P. R. China; 8 Institute of Clinical Pharmacology, Central South University; Hunan Key Laboratory of Pharmacogenetics, Changsha 410078, Hunan, P. R. China

**Keywords:** PDI family, PDI, GBM, glioma, prognosis

## Abstract

The present study focused on the expression patterns, prognostic values and potential mechanism of the PDI family in gliomas. Most PDI family members’ mRNA expressions were observed significantly different between gliomas classified by clinical features. Construction of the PDI signature, cluster and risk score models of glioma was done using GSVA, consensus clustering analysis, and LASSO Cox regression analysis respectively. High values of PDI signature/ risk score and cluster 1 in gliomas were associated with malignant clinicopathological characteristics and poor prognosis. Analysis of the distinctive genomic alterations in gliomas revealed that many cases having high PDI signature and risk score were associated with genomic aberrations of driver oncogenes. GSVA analysis showed that PDI family was involved in many signaling pathways in ERAD, apoptosis, and MHC class I among many more. Prognostic nomogram revealed that the risk score was a good prognosis indicator for gliomas. The qRT-PCR and immunohistochemistry confirmed that P4HB, PDIA4 and PDIA5 were overexpressed in gliomas. In summary, this research highlighted the clinical importance of PDI family in tumorigenesis and progression in gliomas.

## INTRODUCTION

Gliomas are the most common brain and central nervous system (CNS) tumors. According to cancer statistics, gliomas account for approximately 80% of all malignant primary brain tumors and 57.3% of gliomas was glioblastoma (GBM) [1, 2]. Hideo Nakamura et al. reported that the incidence rate for glioma was 6·6 per 100,000 and nearly half of this was glioblastoma [3]. Of note, the Central Brain Tumor Registry of the United States (CBTRUS) statistical report revealed that the incidence rate of glioblastoma increased with age, with 1.24 per 100,000 population in age 35–44 years, 8.07 in age 55–64 years, 12.98 in age 65–74 years and with the highest rates in 75–84 years (15.29 per 100,000 population) [2]. According to WHO classification, GBM, is defined as a grade IV glioma with its molecular classification revealing four subtypes; proneural, neural, classical, and mesenchymal [4, 5]. After a safe surgical resection, GBM patients are treated with radiotherapy and chemotherapy [6]. Unfortunately, their survival rate is very limited with most of the patients dying within 15–18 months post-diagnosis while just 6.8% of the patients survive up to five years [2, 7–9]. Consequently, glioblastoma is a major threat to human health, especially in the elderly.

Protein disulfide isomerase (PDI) family consists of twenty-one members sharing a common structure (the TRX-like domain). Their proteins have an N-terminal signal peptide consisting of 15–30 amino acids which are cleaved upon entry into the endoplasmic reticulum [10]. The PDI family members are mainly located in the ER, with a few also found in other cellular compartments, including the cell surface, cytosol, mitochondria, and nucleus [11]. This PDI family of enzymes have been identified primarily as reductases, oxidases, isomerases, and enzymatic chaperones catalyzing disulfide bond formation, breakage, rearrangement, and oxidative protein folding [11].

Several studies have linked protein disulfide isomerases (PDIs) to the regulation of proliferation, invasion, and metastasis of various cancers, such as brain, lymphoma, kidney, ovarian, prostate, and lung cancers [12]. PDIA6 was found to be over-expressed in bladder cancer (BC) where it facilitated proliferation and invasion in BC cells via Wnt/β-catenin signaling pathway [13]. Moreover, P4HB, PDIA3, and PDIA4 were found to be up-regulated in ovarian cancer and associated with the tumor grade and poor prognosis [14]. PDIA1 and PDIA3 serve vital roles in the progression of diffuse glioma. P4HB function using bacitracin attenuated the phosphorylated FAK and the secreted MMP-2, the downstream molecules of integrin, restrained migration of U87-MG Glioma cells [15]. Other studies have revealed that PDIs inhibition suppresses the growth of tumors. Inhibition of PDIA1 was shown to decrease the resistance to temozolomide (TMZ) in malignant glioma [16, 17]. Recently, Horibe et al. suggested that PDIA5 knockdown in GBM cells significantly suppressed the growth and migration of tumor cells [18].

PDIs have been associated with the progression of GBM, however little is known on the relationship between PDIs expression and the clinical outcome in gliomas. In the present study, the aim was to conduct a comprehensive analysis of twenty-one PDI family members. Bioinformatic tools and qRT-PCR were used for mRNA expression in gliomas. Therefore, data obtained revealed several new potential signaling pathways of PDIs involved in glioma progression. Furthermore, a prognostic nomogram using PDIs-based survival risk score and other clinical factors was established, and these provided novel insights for future diagnostics and therapeutic targets of glioma.

## RESULTS

### The relationship between PDIs mRNA expression and clinicopathological characteristics of glioma

**Figure 1:**
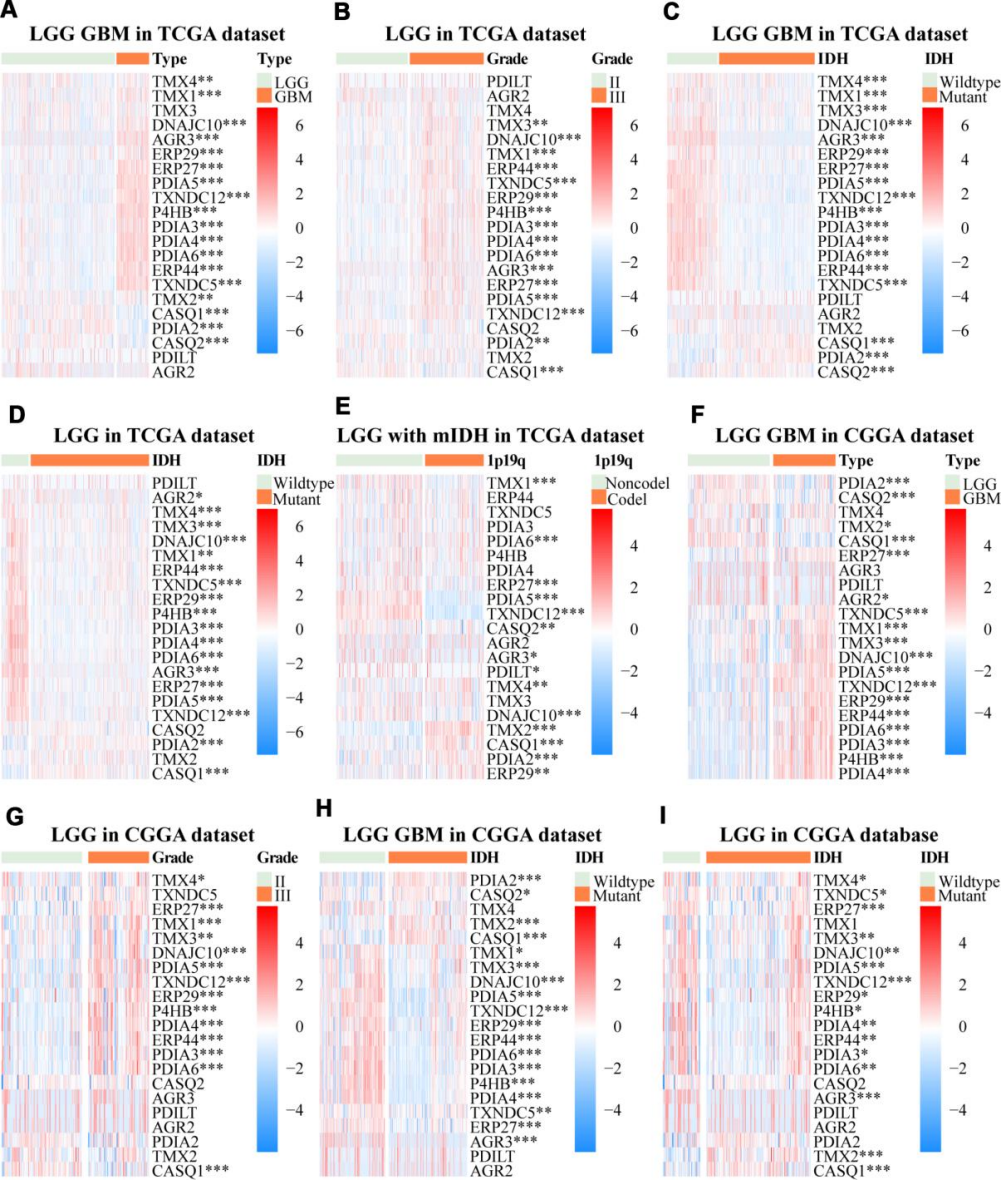
**Relationship between mRNA expression patterns of PDIs in gliomas with different clinical characteristics (cancer type, WHO grade, the status of IDH and 1p19q).** The heat maps, based on the public data from TCGA and CGGA databases, demonstrated upregulated mRNA (red) or downregulated mRNA (blue) of the PDI family members in the subgroups. TCGA database as training set and CGGA database as the validation set. * *p* < 0·05, ** *p* < 0·01, *** *p* <0·001.

In humans, twenty-one members of the PDI family have been identified. In this study, TCGA and CGGA databases data revealed the correlation between PDIs mRNA expression and clinical-pathological features in gliomas. High transcription levels of P4HB, PDIA3, PDIA4, PDIA5, PDIA6, ERP27, ERP29, ERP44, TMX1, TMX3, TXNDC5, TXNDC12, AGR3, and DNAJC10 were found in GBM tissues. However, the transcription levels of PDIA2, AGR2, CASQ1, and CASQ2 were lower in GBM tissues than in LGG tissues (Figure 1A–1F). There was no significant difference in the mRNA level of PDILT between GBM and LGG samples based on both TCGA and CGGA (Figure 1A–1F). However, there were inconsistent findings on the mRNA expression levels of TMX2 and TMX4 from TCGA and CGGA datasets in GBM versus in LGG tissues (Figure 1A–1F). Furthermore, TCGA and CGGA datasets confirmed the significant correlation between WHO grades and mRNA levels of P4HB, PDIA3, PDIA4, PDIA5, PDIA6, ERP27, ERP29, ERP44, TMX1, TMX3, TXNDC12, DNAJC10, and CASQ1 (Figure 1B–1G). In the TCGA LGGGBM cohort, the mRNA expression of P4HB, PDIA3, PDIA4, PDIA5, PDIA6, ERP27, ERP29, ERP44, TMX1, TMX3, TMX4, TXNDC5, TXNDC12, AGR3, and DNAJC10 in gliomas with mutant IDH were lower in comparison to those in gliomas with wild-type IDH. The mRNA expression of PDIA2, CASQ1, and CASQ2 was higher in the mutant IDH group (Figure 1C). There was no significant difference in the expression levels of PDILT, TMX2, and AGR2 between the two groups. Unlike previous findings, within the CGGA LGGGBM cohort, TMX2 was up-regulated in gliomas with mutant IDH and the expression of TMX4 in the two groups was not statistically significant (Figure 1H). There was a significant difference in the expression of some members of the PDI family between the two groups (mutant IDH vs. wildtype IDH) in the LGG and GBM cohort (Figure 1D, 1I, Supplementary Figure 1A, 1B). Further, PDIA2, TMX2, and CASQ1 were down-regulated in LGG with mutant IDH and 1p19q noncodeletion, and PDIA5, PDILT, ERP27, TMX1, TXNDC12, AGR3, and DNAJC10 were up-regulated in both TCGA and CGGA (Figure 1E, Supplementary Figure 1C). There was no significant difference in the mRNA level of other members of the PDI gene family in the low-grade gliomas with mutant IDH (1p19q codeletion vs. 1p19q noncodeletion) in TCGA or CGGA (Figure 1E, Supplementary Figure 1C).

### Construction of the PDI signature model

**Figure 2:**
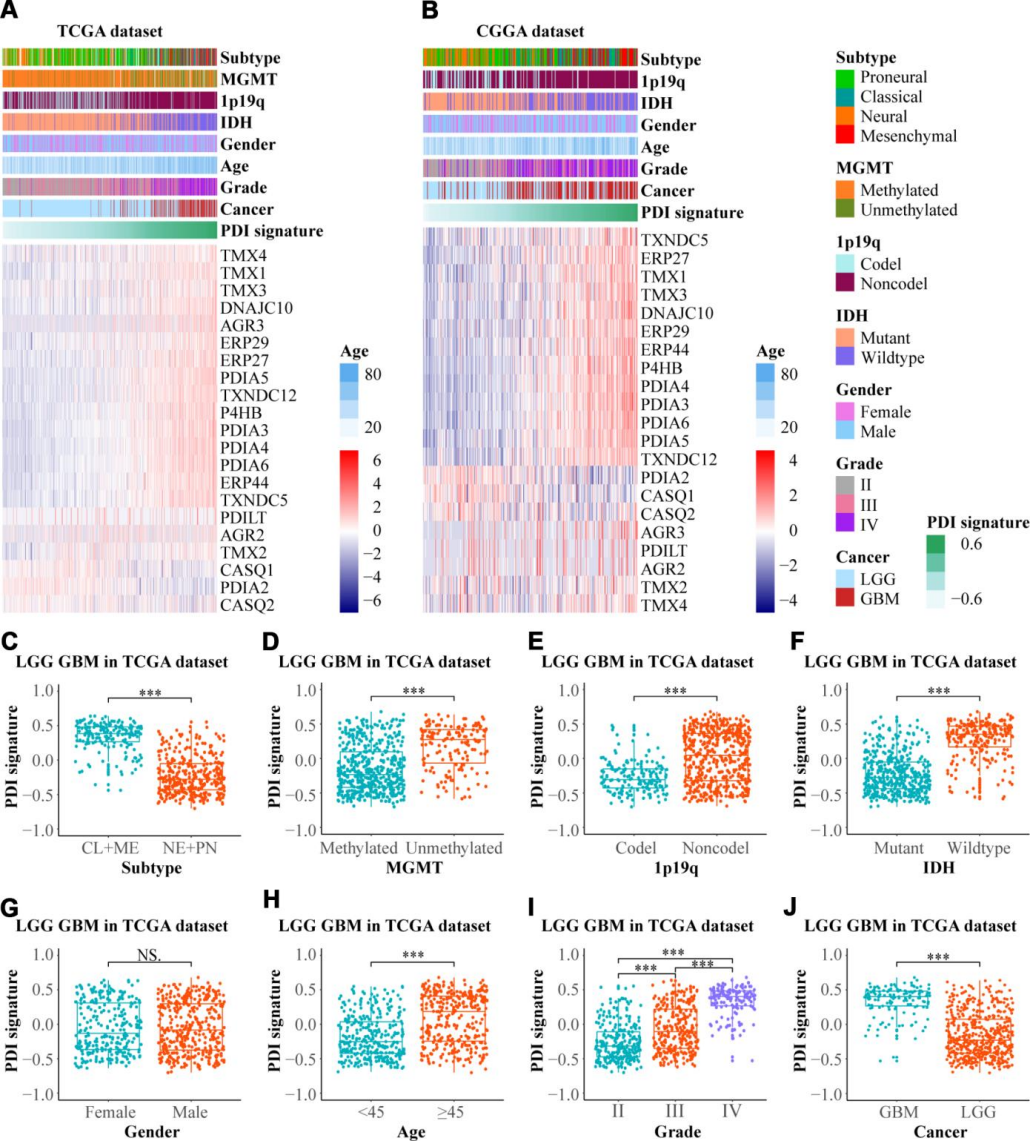
**The relationship between the PDI signature and clinical features in gliomas.** Heat maps revealed the expression profiles of PDIs and the distribution of clinicopathological features in gliomas based on data from TCGA (**A**) and CGGA (**B**) in which the samples were ranked according to their PDI signature. In the TCGA dataset, the distribution of PDI signature in the subgroups classified by subtype (**C**) MGMT promoter status (**D**) 1p19q codel status (**E**) IDH status (**F**) gender (**G**) age (**H**) grade (**I**) and cancer (**J**). TCGA database as training set and CGGA database as the validation set. *** *p* < 0·001, NS. *p* > 0.05.

To construct a PDIs-based signature model for both in training and validation groups GSVA was performed. Heat maps presented the expression profiles of PDI family members ranked according to their PDI signatures from the TCGA and CGGA datasets (Figure 2A, 2B). In the TCGA database, gliomas were classified into four molecular subtypes; proneural (PN), neural (NE), classical (CL), and mesenchymal (ME). In the current study, gliomas were further classified into two main subtypes based on their malignancy (CL+ME vs. NE+PN). The value of PDI signature in patients separated by subtype, MGMT promoter status, 1p19q codel status, IDH status, gender, age, grade, and cancer (LGG vs. GBM). In the TCGA LGGGBM cohort there were significant differences between the patients separated by subtype (CL+ME vs. NE+PN), MGMT promoter status, 1p19q codel status, IDH status, age, grade, cancer (LGG vs. GBM), but not by gender (Figure 2C–2J). Supplementary Figure 1D showed that there was no significant difference in PDI signature between classical and mesenchymal subtypes. Further, there were statistical differences observed in the groups divided by subtype (CL+ME vs. NE+PN), 1p19q codel status, IDH status in TCGA LGG and/or GBM cohort. However, there was no significant difference in the MGMT promoter status and IDH status in the TCGA GBM cohort (Supplementary Figure 1E–1J).

The patients were divided into two groups (high vs. low group) using the median value of PDI signature as the cut-off value to investigate the relationship between the value of PDI signature and patients’ prognosis. In the TCGA LGGGBM cohort, the Kaplan–Meier plot revealed that the high value of PDI signature was associated with poor OS, PFI and DSS (Supplementary Figure 2A–2C). Similar findings were also found in LGG and GBM (Supplementary Figure 2D–2I). Furthermore, as validated in the CGGA datasets, patients in the low-value group exhibited longer OS than those in the the high-value group (Supplementary Figure 2J–2L). These findings indicated a significant association between PDI signature and clinical features and the high value of PDI signature was associated with poor prognosis.

**Figure 3:**
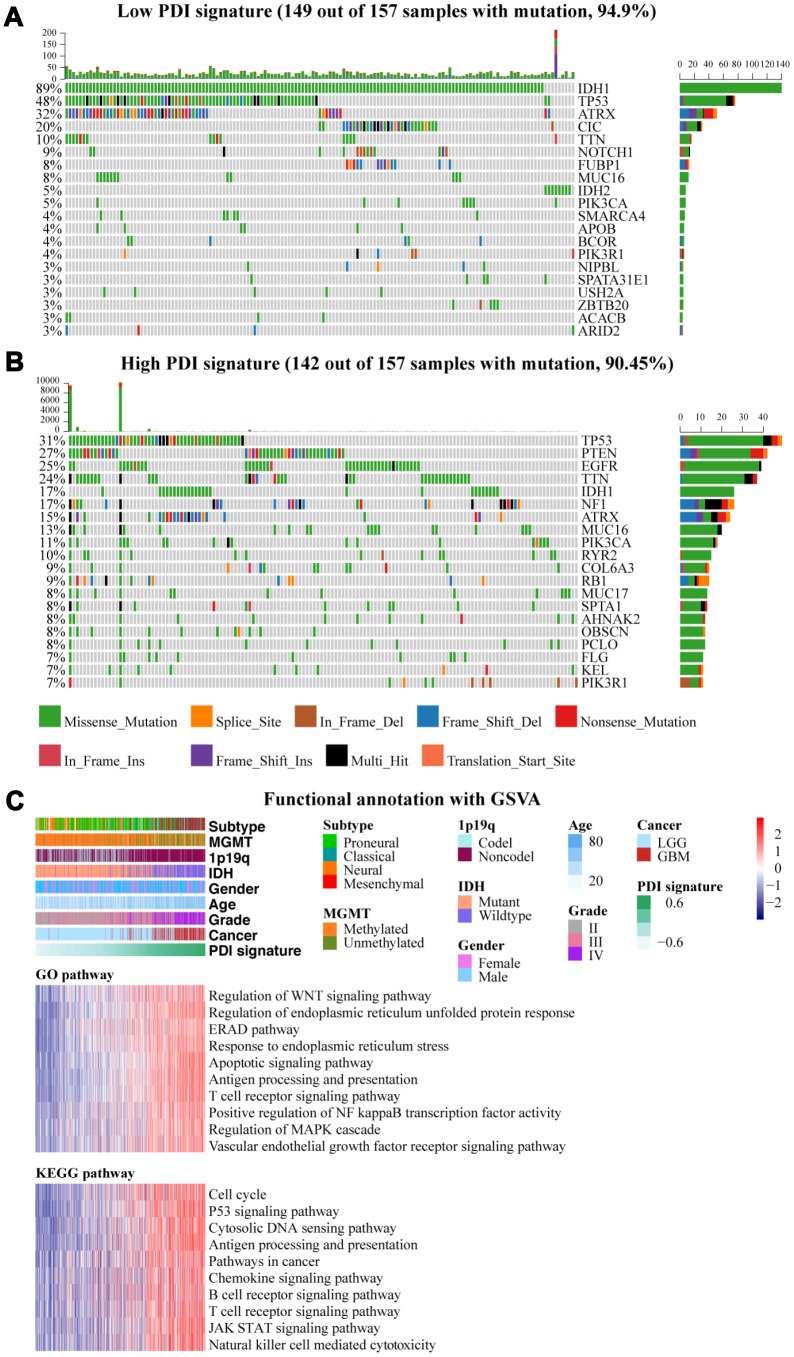
(**A**, **B**) Genetic alteration profiles associated with PDI signature in TCGA and CGGA datasets. Oncoprint depicts the distribution of the top 20 genes with the highest mutation frequency in each glioma group (low PDI signature vs high PDI signature). (**C**) Functional annotation of PDI gene family with PDI signature, including GO and KEGG. The upper one panel shows the distribution of PDI signature and clinical features, and the lower two panels show the gene set enrichment in different pathways analyzed by GSVA package of R. TCGA database as training set and CGGA database as the validation set.

As previously described, somatic mutations and copy number variations in the two groups were analyzed (1^st^ vs. 4^th^). High mutation frequency in IDH1, TP53, and ATRX were associated with low PDI signature in gliomas (IDH1, 89% vs. 17%; TP53, 48% vs. 31%; ATRX, 32% vs. 15%), whereas TTN, MUC16, and PIK3CA were associated with high PDI signature (TTN, 10% vs. 24%; MUC16, 8% vs. 13%; PIK3CA, 5% vs. 11%) (Figure 3A–3B). The mutation frequency of CIC in the low PDI signature group reached 20% (Figure 3A) while the mutations in PTEN, EGFR, NF1, and RYR2 were enriched in the cases with high PDI signature, of which all their frequencies were more than 10% (Figure 3B).

GSVA was performed to characterize the potential function of PDIs in gliomas. Most of the functional pathways were enriched in higher PDI signature group. Some important biological processes in which PDI was known to be involved were identified, such as unfolded protein response (UPR), endoplasmic reticulum-associated degradation (ERAD), endoplasmic reticulum stress (ERS). Many pathways were also associated with cancer pathogenesis, invasion, and metastasis, including WNT signaling pathways, positive regulation of NF kappa B transcription factor activity, vascular endothelial growth factor receptor (VEGFR) signaling pathway, apoptotic signaling pathway, regulation of MAPK cascade, cell cycle, p53 signaling pathway, cytosolic DNA sensing, pathways in cancer, and JAK-STAT signaling pathway. There were also immune-related regulatory pathways identified which did not traditionally focus on PDIs such as antigen processing and presentation, chemokine signaling pathway, B cell receptor signaling pathway, T cell receptor signaling pathway, and natural killer cell-mediated cytotoxicity (Figure 3C).

### Constructing the cluster model of glioma based on consensus clustering analysis

**Figure 4:**
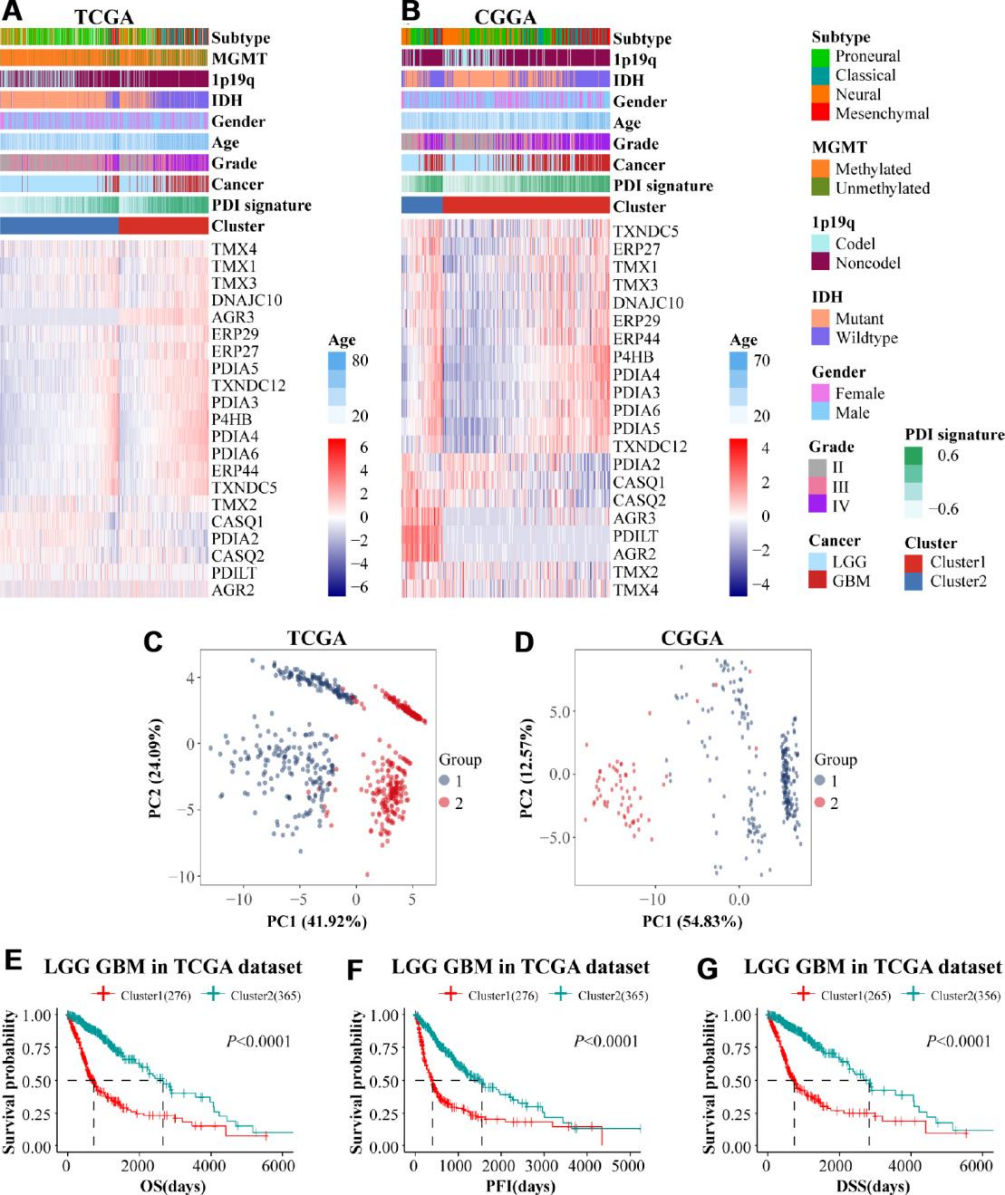
(**A**, **B**) Comparison of the expression levels of PDIs and clinical features between two subgroups (cluster 1 vs cluster 2). Principal component analysis (PCA) was performed to reveal the difference of PDIs mRNA expression between two clusters in TCGA (**C**) and CGGA (**D**). The prognostic value of clusters in glioma patients. Kaplan–Meier survival analyses were used to demonstrate differences in OS, PFI and DSS between the two clusters of LGGGBM samples from TCGA (**E**–**G**) and CGGA (**H**). TCGA database as training set and CGGA database as the validation set.

Tumor samples from TCGA and CGGA datasets were grouped into two clusters (cluster 1, cluster 2) using consensus clustering analysis to explore PDIs’ potential predictive and prognostic value. Cumulative distribution function (CDF) curves and consensus matrixes (Supplementary Figure 3A–3D) determined the optimal number of clusters (k=2). As shown in Figure 4A–4B, cluster 1 was related to the status of MGMT promoter unmethylated, 1p19q noncodel, IDH wildtype, higher grade, and GBM. Glioma patients in cluster 1 also had a higher PDI signature compared with cluster 2. The principal component analysis demonstrated the difference of PDIs mRNA expression between the two clusters (Figure 4C–4D).

Apart from differences in clinical characteristics between cluster 1 and cluster 2, there were also differences in prognosis in the two groups. In the TCGA datasets, Cluster 2 significantly correlated with longer OS, PFI and DSS in contrast to cluster 1 (Figure 4E–4G).

### Constructing the risk score model based on least absolute shrinkage and selection operator (LASSO) Cox regression analysis

**Figure 5:**
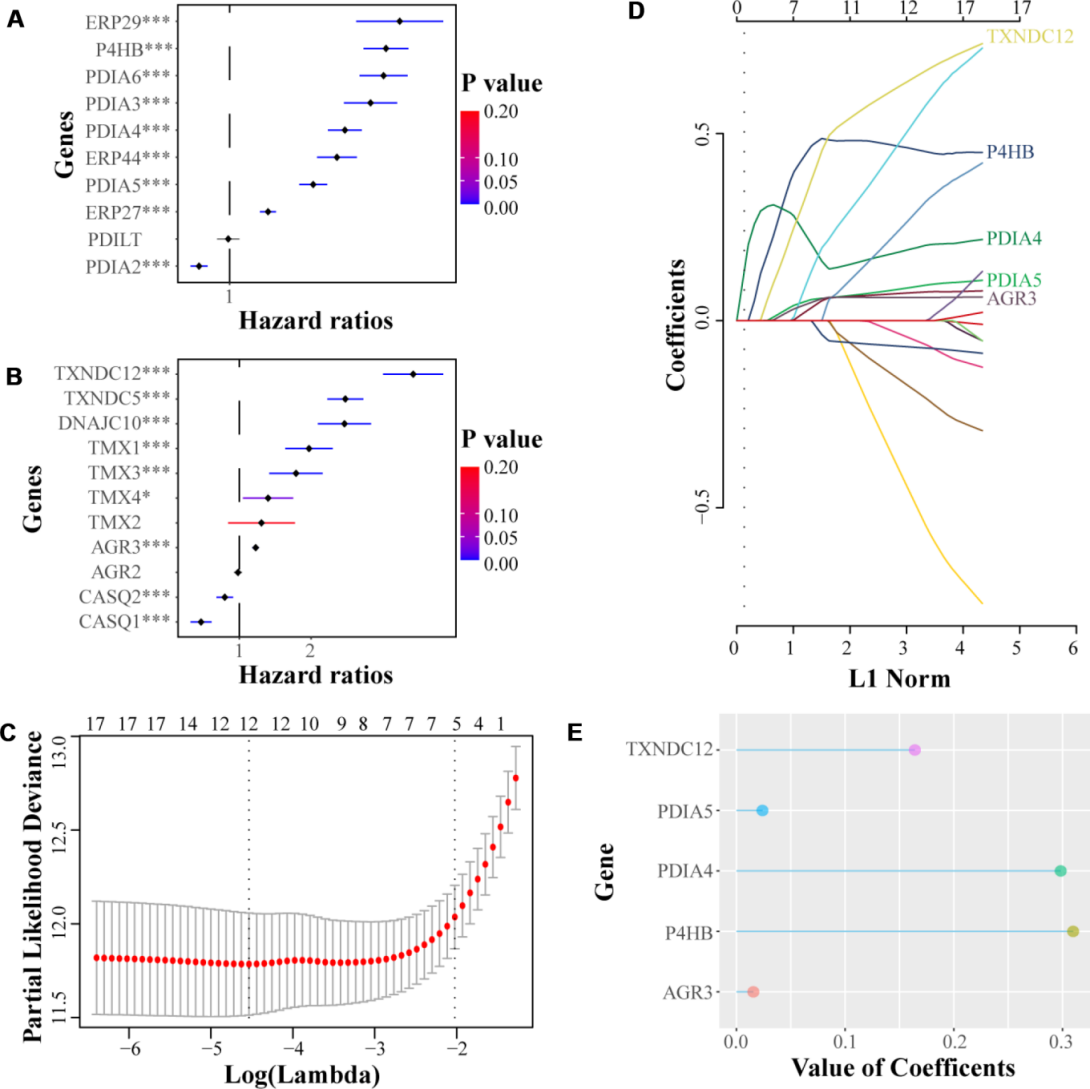
(**A**, **B**) Univariate Cox regression analyses were performed to select significant genes from PDI family according to the clinical information from TCGA. (**C–E**) LASSO coefficients of the significant members of PDI family for OS were calculated, of which the five most influential ones are presented in the figure. * *p* < 0·05, *** *p* < 0·001.

**Figure 6:**
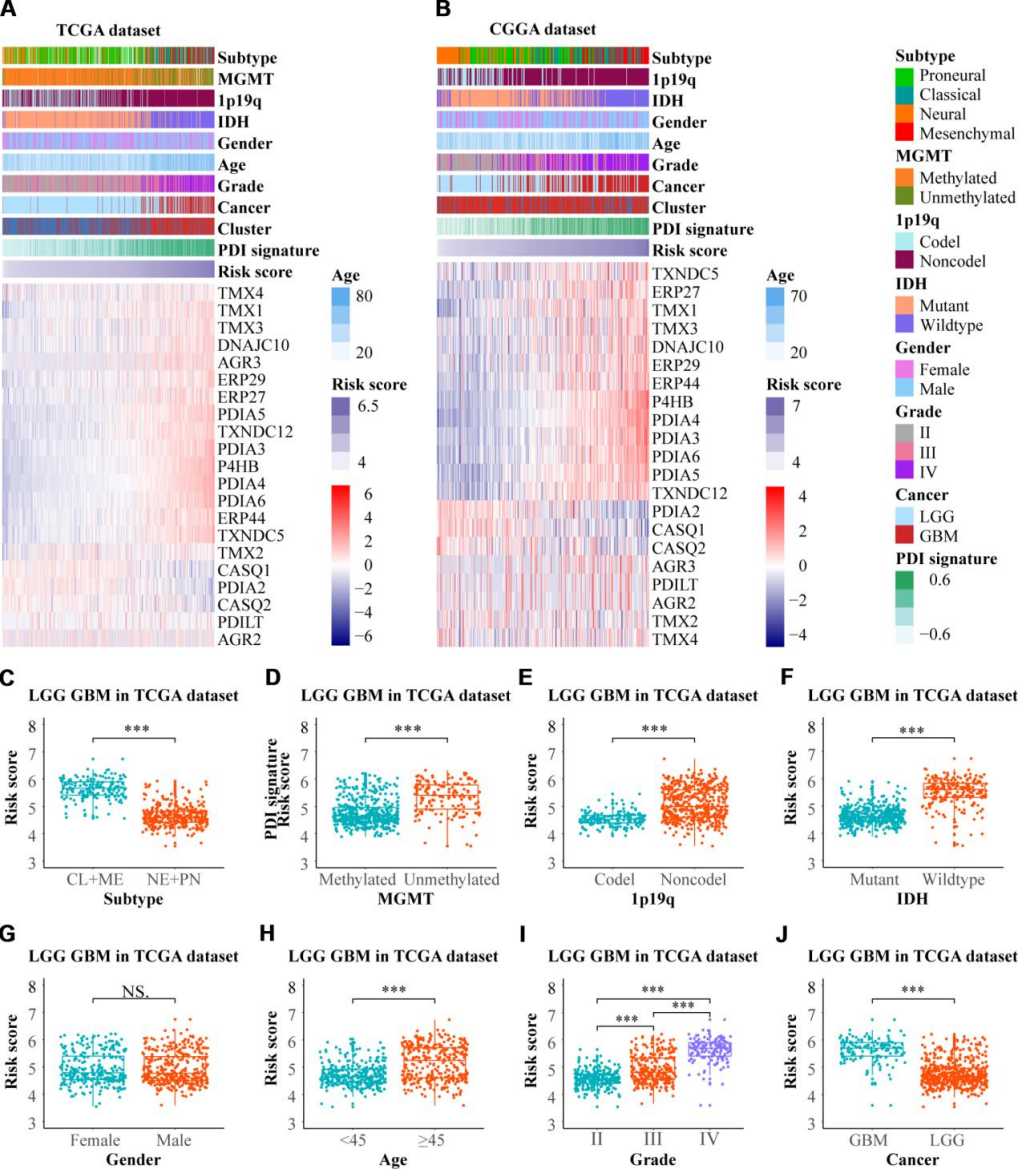
(**A**, **B**) The risk score model of PDI family in gliomas was established on the basis of LASSO coefficients. The distributions of clinical features and PDIs expression according to the risk scores from low to high in TCGA and CGGA are displayed by heat maps. (**C–J**) The risk scores differences between subgroups classified by subtype, MGMT promoter status, 1p19q codel status, IDH status, gender, age, grade, and cancer in LGGGBM cohort. TCGA database as training set and CGGA database as the validation set. *** *p* < 0·001, NS. *p* > 0·05.

A total of 18 significant factors with *p* < 0.05 were selected through the univariate COX regression analysis (P4HB, PDIA2, PDIA3, PDIA4, PDIA5, PDIA6, ERP27, ERP29, ERP44, TMX1, TMX3, TMX4, TXNDC5, TXNDC12, AGR3, DNAJC10, CASQ1, CASQ2) (Figure 5A–5B). These were then introduced into the LASSO Cox regression model, which exhibited the five most meaningful PDI family members and their coefficients which was used to calculate the risk score (Figure 5C–5E). The heat maps displayed the expression profiles of PDIs both in TCGA and CGGA datasets, as the samples were ranked according to their risk scores (Figure 6A, 6B). In the TCGA LGGGBM dataset, the risk score in different groups was stratified by clinical features, and exhibited by box plots. The higher risk score was related to the subtype (CL+ME vs. NE+PN), MGMT promoter unmethylated, 1p19q noncodel, IDH wildtype, age≥45, and GBM groups (Figure 6C–6F, 6H, 6J, 6E). As shown in Supplementary Figure 3E, there was no significant difference in the risk score between classical and mesenchymal subtypes. Moreover, the risk score increased along with the WHO grade of gliomas (Figure 6I). However, there was no significant difference in groups separated by gender (Figure 6G). There were statistical differences in the LGG/GBM patients classified by subtype (CL+ME vs. NE+PN), 1p19q codel status, IDH status, but not by MGMT promoter status in the TCGA GBM cohort (Supplementary Figure 3F–3K).

**Figure 7:**
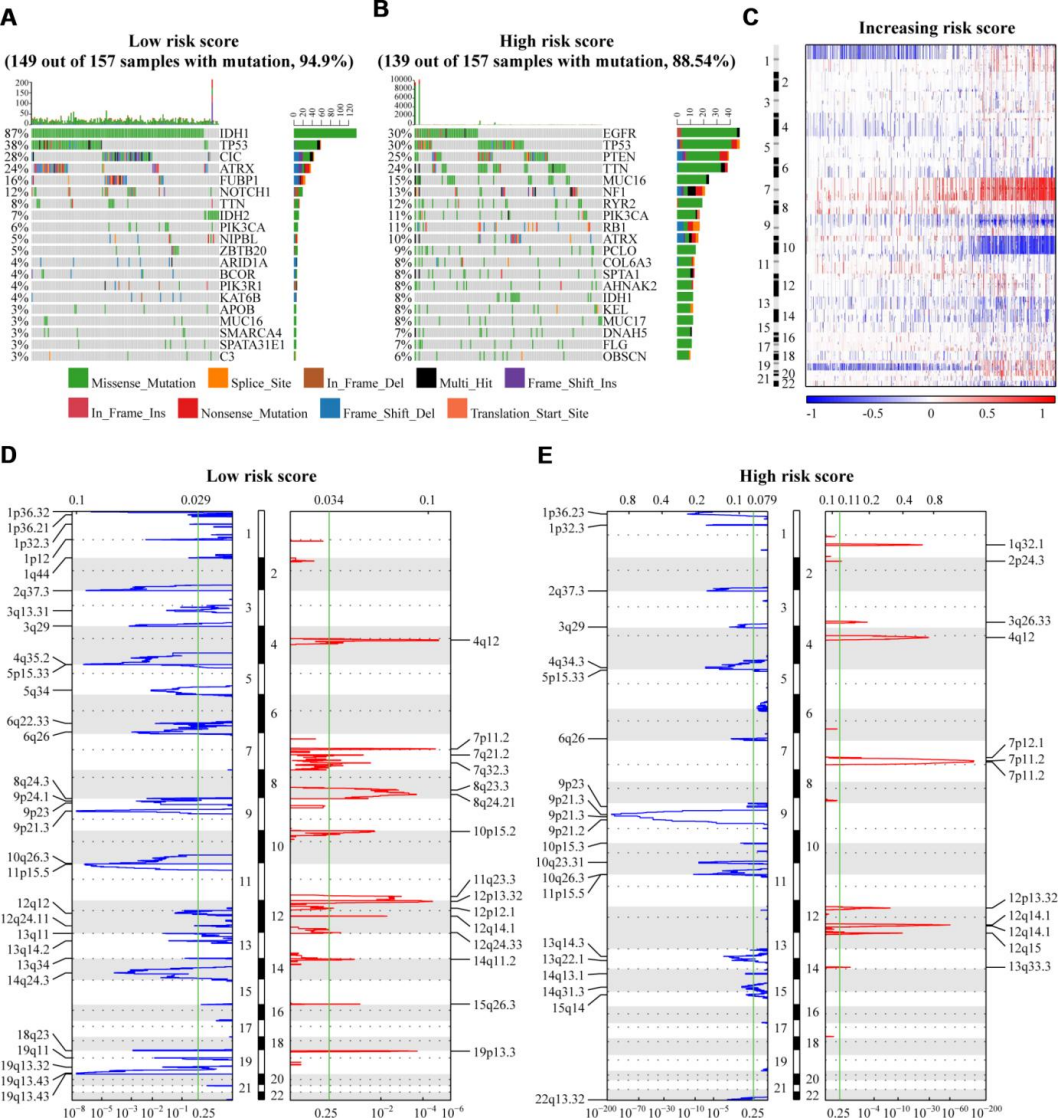
(**A**, **B**) The significantly mutated genes in gliomas were assigned to low and high risk score groups. Here only the top 20 genes with the highest mutation frequency are displayed in figures. (**C**) The overall CNAs profile in order of increasing risk score. (**D–E**) GISTIC 2.0 analysis of cases with low and high risk scores revealed chromosomal regions that were significantly deleted (blue) and amplified (red). The green line represents the significance threshold (*q* value=0·25). TCGA database as training set and CGGA database as the validation set.

The prognostic differences between low and high-risk groups divided by the median risk score were compared. As shown in Supplementary Figure 4A–4I, the patients with high-risk score had significantly shorter OS, PFI, and DSS than those with a low-risk score in the TCGA LGGGBM, LGG, GBM cohorts. Similarly, the high-risk score was correlated with poor OS in the CGGA datasets (Supplementary Figure 4J–4L). In summary, these findings imply that the PDIs-based model plays an important role in the prediction of patients’ clinicopathological characters and prognosis. Somatic mutations were observed in 149 (94.9%) and 139 (88.54%) of 157 samples in the groups with a low and high-risk score respectively. There were some genes shown mutations in the two groups (low vs. high- risk score): IDH1, TP53, ATRX, TTN, PIK3CA, and MUC16. The frequency of IDH1, TP53, and ATRX mutations was significantly higher in gliomas with low risk score than those with high risk score (IDH1, 87% vs. 8%; TP53, 38% vs. 30%; ATRX, 24% vs. 10%), while the mutation frequency of TTN, PIK3CA, and MUC16 was significantly lower (TTN, 8% vs. 24%; PIK3CA, 6% vs. 11%; MUC16, 3% vs. 15%). Mutations of CIC (28%), FUBP1 (16%), and NOTCH1(12%) in low risk score group and, EGFR (30%), PTEN (25%), NF1 (13%), RYR2 (12%), and RB1 (11%) in high risk score group were identified (Figure 7A, 7B).

SCNAs between samples with low and high-risk scores was investigated considering the somatic copy number alternations’ roles in oncogenesis. The incidence of the amplification of Chr 7 together with a deletion of Chr 10 increased with an increase in the value of risk score in gliomas, while the codeletion of 1p/19q decreased (Figure 7C). GISTIC 2.0 analysis identified several significant regions of amplification harboring multiple oncogenes in gliomas with a high-risk score, including 1q32.1 (PIK3C2B), 4q12 (PDGFRA), 7p11·2 (EGFR), and 12q14·1(CDK4). Focal deletion peaks were detected in the group with a high-risk score, such as 9p21·2 (TUSC1), 9p21·3 (CDKN2A, CDKN2B), 10q23·31 (PTEN, FAS), and 10q26·3 (BNIP3). Although the focal amplification and deletion peaks were also present in the low risk score cases, their G values were significantly lower (Figure 7D–7E). Additionally, there were significant genomic regions of amplification (8q23·3, 8q24·21, 11q23·3, 19q13·3) and deletion (1p12, 4q35·2, 14q24·3, 18q23, 19q13·43) observed only in gliomas with low-risk scores (Figure 7E).

**Figure 8:**
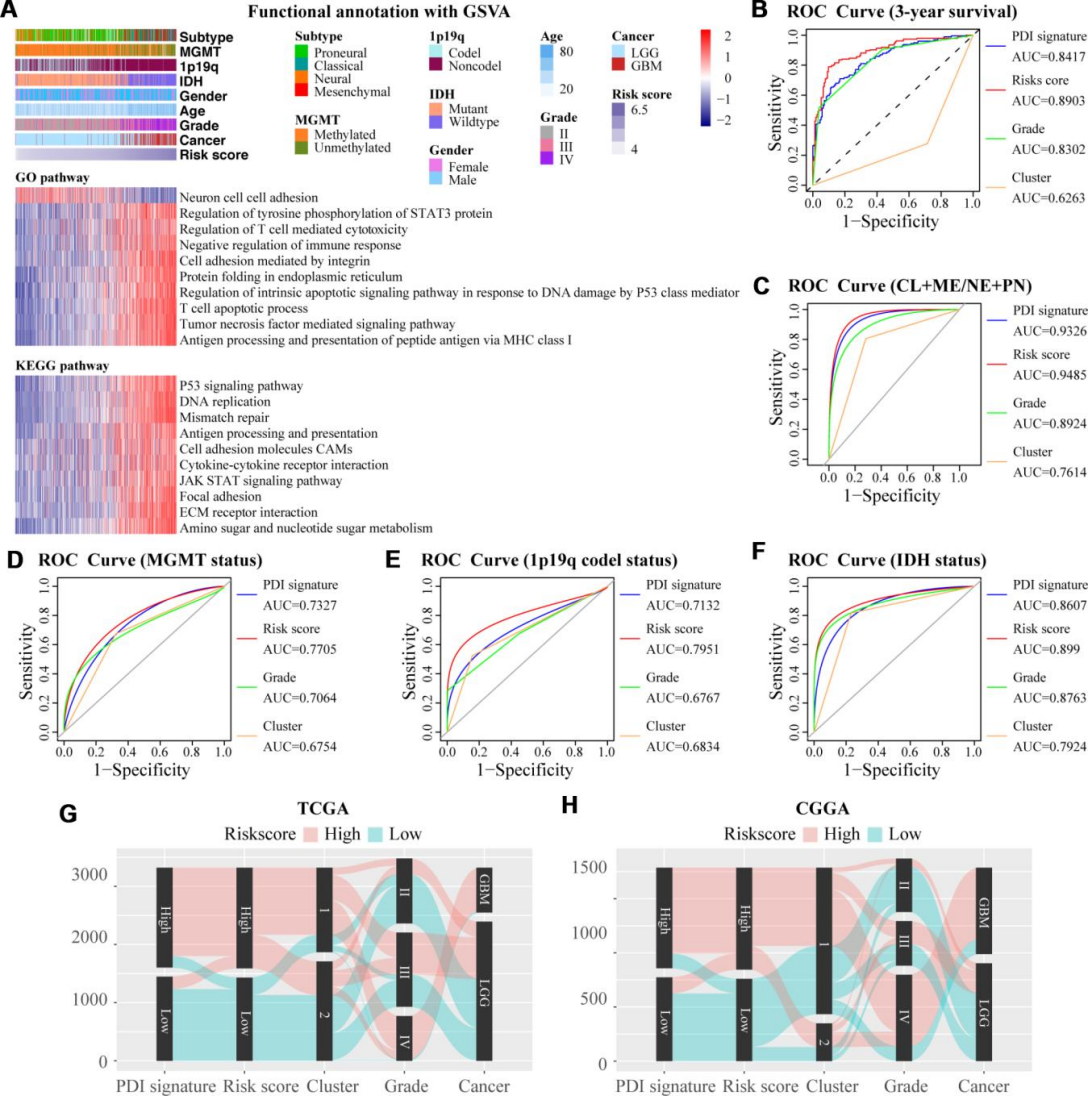
(**A**) GO and KEGG analyses for the PDI gene family and the risk scores determined using GSVA. The heat map shows the distribution of risk scores and clinical features (upper one panel), and gene set enrichment of different pathways (lower two panels). Comparisons of the performance of PDI signature, risk score, grade and cluster in predicting the 3-year overall survival of glioma patients (**B**) subtype (**C**) MGMT promoter status (**D**) 1p19q codel status (**E**) and IDH status (**F**). (**G**, **H**) The relationship among the five indicators, PDI signature, risk score, cluster, WHO grade, and cancer type. TCGA database as training set and CGGA database as the validation set.

The GSVA analysis identified the biological functions of the PDI gene family in gliomas. From the GO and KEGG enrichment analysis, 10 signaling pathways having statistical significance and high correlation coefficient were selected. The higher gene set enrichment scores of all the selected pathways were associated with higher risk scores, excepting for neuron cell-cell adhesion. Some pathways were related to immune response, including T cell apoptotic process, antigen processing and presentation of peptide antigen via MHC class I, regulation of T cell-mediated cytotoxicity, negative regulation of immune response, cytokine-cytokine receptor interaction, and antigen processing and presentation. The cell adhesion-related pathways were also enriched, including neuron cell-cell adhesion, cell adhesion mediated by integrin, cell adhesion molecules CAMs, and focal adhesion. Biological processes significantly associated with the mechanisms of PDIs in gliomas included protein folding in endoplasmic reticulum, regulation of intrinsic apoptotic signaling pathway in response to DNA damage by P53 class mediator, tumor necrosis factor-mediated signaling pathway, regulation of tyrosine phosphorylation of STAT3 protein, ECM receptor interaction, JAK-STAT signaling pathway, amino sugar and nucleotide sugar metabolism, P53 signaling pathway, DNA replication, and mismatch repair (Figure 8A).

### Comparisons among PDI signature, cluster, risk score and grade in predicting prognosis and clinical features

ROC analysis compared the PDI signature, risk score, cluster and grade in predicting prognosis and clinical characters. The risk score was the best indicator in predicting 3-year survival, subtype (CL+ME/NE+PN), MGMT status, 1p19q codel status and IDH status. Consensus clustering analysis model was the least performer in predicting prognosis and clinical features (Figure 8B–8F). The Sankey diagrams indicated that glioma patients with high-risk scores had higher PDI signature, higher grade and mainly enriched in cluster1, while the low-risk score was associated with low PDI signature, lower grade and cluster2 (Figure 8G–8H). Besides, there was a positive correlation between risk score and PDI signature (r = 0·84, *p* < 0.001) (Supplementary Figure 5A).

### Constructing prognostic nomogram for overall survival

**Figure 9:**
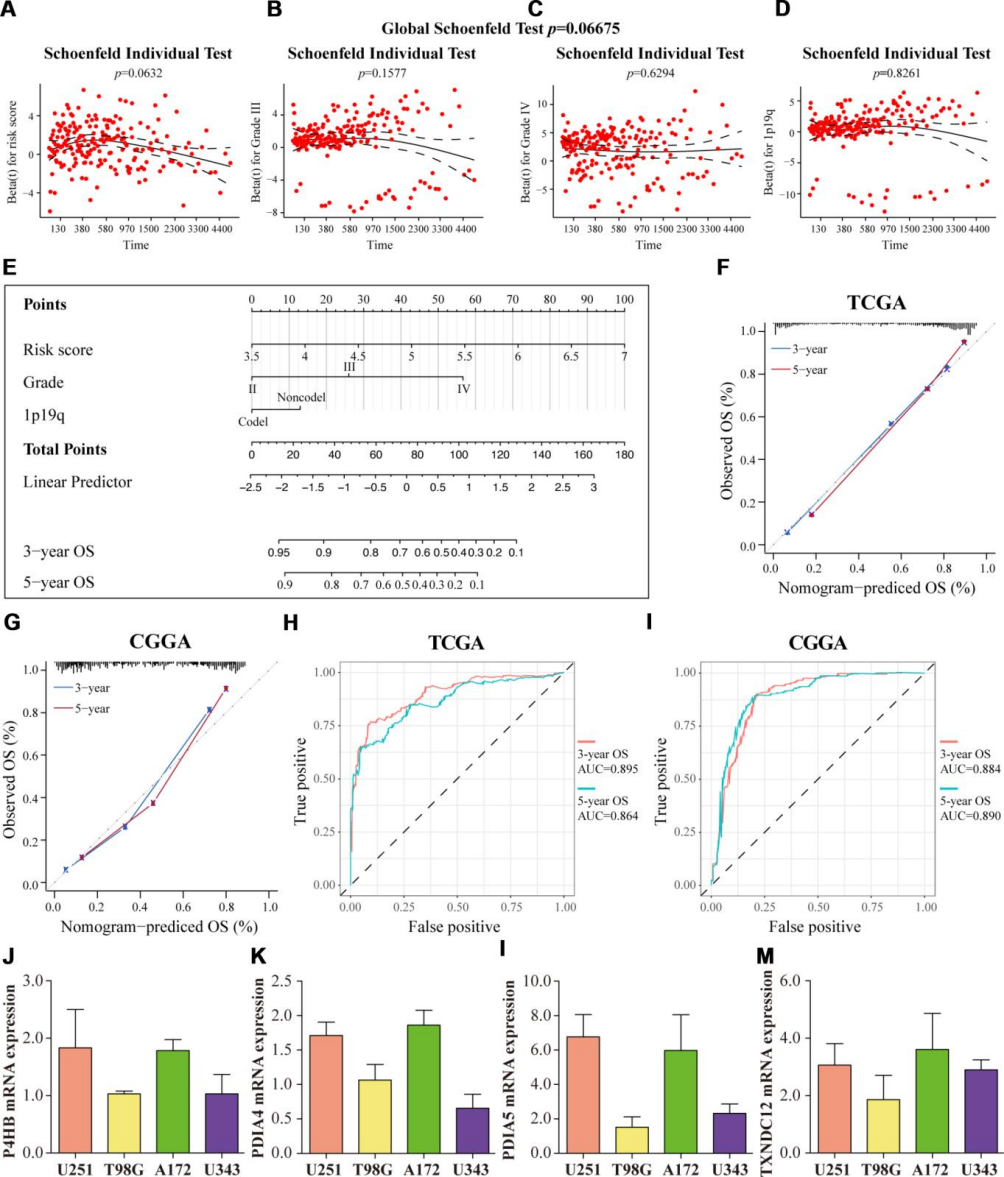
**Prognostic nomogram for predicting the 3-year and 5-year overall survival of glioma patients.** The Schoenfeld residual plots were displayed for risk score (**A**) Grade III (**B**) Grade IV (**C**) 1p19q (**D**) in the prognostic nomogram. The solid line was a smoothing-spline fit to the plot, with the dashed lines representing the 95% confidence interval. (**E**) Prognostic nomogram for glioma patients was created based on four key characteristics. (**F**, **G**) The calibration curve of OS at 3 years (blue) and 5 years (red). The predicted probability of OS is plotted on the x-axis and the observed OS is plotted on the y-axis. (**H**, **I**) ROC curves from the nomogram of 3-year and 5-year OS. The mRNA expression patterns of P4HB (**J**) PDIA4 (**K**) PDIA5 (**L**) and TXNDC12 (**M**) in glioma cell lines (U251, T98G, A172, U343). TCGA database as training set and CGGA database as the validation set.

Univariate and multivariate Cox regression analyses using clinical data downloaded from the TCGA and CGGA database investigated the independent prognostic indicators related to patients' clinical outcomes (OS, DSS, PFI). Risk score (TCGA *p* = 0·0198, HR = 1·66; CGGA *p =* 0·0072, HR = 1·56) and 1p19q (TCGA *p* = 0·0392, HR = 1·84; CGGA *p =* 2·35E-05, HR = 3·27) were independent prognostic indicators for OS time. Considering parameters of grade III and grade IV with statistical significance, the grade was adopted as one of the prognostic indicators as well (Supplementary Table 1). As shown in Figure 9A–9D, the *P* value of the global Schoenfeld test and variables were all greater than 0.05, indicating that the model and each variable were satisfied with the PH (proportional hazards) test (global Schoenfeld test *p* = 0.06675, risk score, *p* = 0·0632; Grade III, *p* = 0·1577; Grade IV, *p* = 0·6294; 1p19q, *p* = 0·8261). There was no statistical significance in IDH and age in the multivariate Cox regression analysis in CGGA datasets (IDH, *p* = 0·1604; age, *p* = 0·5103). Through Cox regression analyses, risk score, WHO grade, IDH, 1p19q, and age were identified as independent factors in predicting DSS, while risk score and IDH were identified as significant prognostic factors of PFI (Supplementary Table 1).

Cox regression model identified three significant prognostic variables which were used to construct the nomogram. Each of the variables was initially assigned a score by finding its position on the corresponding axis. The points of all the variables were added up and the probabilities of the outcomes determined by the location of the total score on the survival axes (Figure 9E). Calibration curves validated the accuracy of the nomogram in TCGA and CGGA and the nomogram-predicted OS corresponded closely with the observed OS at 3- and 5-year OS (Figure 9F–9G). This data prepared the ROC curve and the AUC values were 0·918 and 0·875 in predicting 3-year and 5-year OS for glioma patients in TCGA, respectively (Figure 9H). A similar process was performed in CGGA, and gave an AUC of 0·862 and 0·875 for the 3- and 5-year OS, respectively (Figure 9I).

The prognostic model value was calculated according to the formula, value = *β_1_X_1_*+ *β_2_X_2_* + … + *β_n_X_n_* (*β,* regression coefficient; *X,* prognostic factors). There was a significant difference in the OS between the two groups (*p* < 0·0001) (Supplementary Figure 5B). Similar results were observed in CGGA (*p* < 0·0001) (Supplementary Figure 5C).

### The mRNA and protein expression patterns of PDIs in glioma

The qRT-PCR assay was performed to identify significant genes for construction of the risk score model in gliomas. AGR3 was eliminated as it played a non-significant role in this model. As shown in Figure 9J–9M, all four genes, P4HB, PDIA4, PDIA5, and TXNDC12, were expressed in glioma cell lines, and these genes were differentially expressed in different cell lines (U251, T98G, A172, U343).

Furthermore, the immunohistochemistry-based expression data of PDI gene family members was obtained from the online Human Protein Atlas. As shown in Supplementary Figure 5D–5E, the protein expression of P4HB and PDIA4 were higher in glioma tissues compared to normal brain tissues. Additionally, PDIA5 protein was not detected in normal tissues, and it was lowly expressed in cancer tissues (Supplementary Figure 5F). However, the protein expression pattern of TXNDC12 was not presented here due to lack of immunohistochemistry images.

## DISCUSSION

In the last three decades, the incidence rate of primary malignant brain and other CNS tumor has increased year by year, with an annual rate of 1%-2%, especially in the elderly population [1]. The average annual age-adjusted incidence rate (AAAIR) of GBM was the highest among malignant brain and other CNS tumors (3.22 per 100,000 population) [2]. Recently, CBTRUS also reported that the median age at diagnosis for GBM was 65 years and survival time was markedly reduced in the elderly patients [2]. Therefore, it is necessary to explore the pathogenesis of glioblastoma to save the older patients from this difficult situation. Dorota et al. showed that PDIA6 was over-expressed in migrating glioma cells and invasive glioma cells, indicating the important role PDIA6 plays in glioma invasion [19]. However, Tae-Wan Kim et al. reported that the inhibition of PDIA6 transduced EGFR signaling via activating and inducing ADAM17 enhanced U87MG cell migration and invasion [20]. However, no study the analyzes the their effects of the twenty-one PDI members on the progression and clinical outcome of gliomas. Therefore, in this study, bioinformatics analysis, GSVA, consensus clustering analysis, and LASSO Cox regression analysis, were used to construct PDI signature, cluster and risk score models of glioma, respectively. The efficacy of PDI signature, risk score and cluster in predicting the clinical characteristics and clinical outcomes were analyzed and compared and risk score was the best indicator. Somatic alterations analysis revealed that gliomas with high PDI signature and risk score were associated with genomic aberrations of driver oncogenes (TTN, MUC16, PIK3CA), but had less mutations of IDH1, TP53, and ATRX. Furthermore, amplification peaks of oncogenes (PIK3C2B, PDGFRA, EGFR, CDK4), and deletion peaks of tumor suppressor genes (TUSC1, CDKN2A, CDKN2B, PTEN, FAS, BNIP3) were detected in the gliomas with a high risk score. These findings revealed that the PDI family are involved in the malignant biological process in gliomas.

GSVA analysis investigated the mechanism of PDIs in gliomas. Consistent with previous studies, the common biological functions of PDIs identified in tumorigenesis and development, included unfolded protein response, endoplasmic reticulum-associated degradation, endoplasmic reticulum stress, cell adhesion, apoptosis, WNT signaling pathways, and cytosolic DNA sensing. There is sufficient evidence supporting that PDI family members are considered as potential targets for tumors due to their functions in UPR, ERAD and ERS signaling pathways. Integrins have been confirmed to be associated with glioma cell invasion, migration and temozolomide resistance [21–23]. Thiol-disulfide exchange reactions in integrins, which play a pivotal role in their activation, are regulated by PDI [15, 19, 23]. Previous studies have not only revealed that PDI is located in the endoplasmic reticulum, which restrains ER stress induction by preventing the accumulation of misfolded proteins which inhibits apoptosis but also suggested that cytosolic PDI is a substrate for caspase-3 and -7 which possesses the anti-apoptotic function [12, 24–27]. Further, Kuo et al. demonstrated that PDIA4 inhibited cell death via preventing the activation/degradation of procaspases-3 and -7 to promote tumor growth and metastasis in lung carcinoma [28].

The findings of function annotation revealed a number of unreported signaling pathways in gliomas which PDIs may be involved in and included P53 signaling pathway, cell cycle, tumor necrosis factor (TNF) mediated signaling pathway, regulation of MAPK cascade, JAK-STAT signaling pathway, positive regulation of NF kappa B transcription factor activity, regulation of tyrosine phosphorylation of STAT3 protein, vascular endothelial growth factor receptor signaling pathway, DNA replication, and mismatch repair. Green et al. noted that PDIA3 was able to interact with P53 to inhibit P53-meditated apoptosis [29]. Similarly, there are studies supporting that PDIs contribute to the regulation of the activity of signaling pathways involved in cellular proliferation, apoptosis, and oncogenesis through interaction with targets, such as TNF, STAT3, NF kappa B [30–33].

An important finding was that PDIs were associated with immune regulation in gliomas, such as negative regulation of immune response, antigen processing and presentation of peptide antigen via MHC class I, T cell receptor signaling pathway, T cell apoptotic process, regulation of T cell-mediated cytotoxicity, antigen processing and presentation, chemokine signaling pathway, B cell receptor signaling pathway, and natural killer cell-mediated cytotoxicity. Gastric cancer patients having high expression of PDIA3 had a favorable prognosis and this was associated with the involvement of PDIA3 in antigen processing and formation of a complex with MCH class I which induced an immune response [34]. However, up-regulation of PDIA3 was associated with poor prognosis in gliomas [17]. Previous studies have shown that the major histocompatibility complex class I chain-related protein A (MICA) is shed from tumor cells via PDIA6 reducing the disulfide bond between them, facilitating the escape of tumor cells from immune responses [18, 35]. These findings demonstrated that the immune response could be explored further to understand the pathogenesis of the PDI family in gliomas.

Considering the important roles of PDI family members in cancer progression, some studies have designed PDIs’ inhibitors. Herein, we have briefly reviewed the previously reported inhibitors for PDI family members. Bacitracin, interacting with reduced PDI and then interfering the integrin, inhibits the glioma cell migration and facilitates apoptosis induced by chemotherapy agents in melanoma cells. BAP2 and analogs play key roles in inducing ER stress, decreasing DNA repair proteins’ expression, inhibiting cell migration and growth in GBM via binding to His256 in the b domain of PDI [36]. The compounds, as the inhibitor of P4HB and PDIA2, reduce the expression of DNA damage and repair genes [37]. 35GB, the cytotoxic inhibitor of PDI, makes significant contributions in nuclear factor erythroid-2-related factor 2 (Nrf2) antioxidant pathway, ERS response, and autophagy [38]. Previous studies demonstrated that anacardic acid and ribostamycin are able to enhance the cytotoxic activity of TMZ by inhibiting the reductase and chaperone activities of PDIA6 respectively [39]. Besides, there are other compounds functioning as inhibitors of PDIs in cancers, such as T8, ML359, PACMA31, CCF642 [40–43].

In summary, this study the expression patterns, prognostic value, and potential mechanisms of the PDI gene family in gliomas were analyzed. This study was the first to construct clinical models in predicting prognosis and clinicopathological characteristics of glioma and revealed potential signaling pathways. However, further research should be shed light on the validation of PDIs biological impacts on gliomas.

## MATERIALS AND METHODS

### Datasets analysis

RNA-seq data and corresponding clinical information were downloaded from the TCGA database (http://cancergenome.nih.gov) as training set and from CGGA database (http://www.cgga.org.cn) as the validation set, which was summarized in Supplementary Table 2. Heat maps were then created to visualize differences in PDIs expression.

### Gene set variation analysis (GSVA)

The PDI signature for each glioma sample was established using GSVA, a non-parametric and unsupervised algorithm. The heat maps and box plots were used to illustrate the relationship between PDI signature and PDIs mRNA expressions and clinical features of gliomas. The functional enrichment analysis was performed using “GSVA package” to reveal the potential signaling pathways of PDIs involved in glioma, setting |correlation coefficient| > 0·5 as the cutoff value [44]. Gene Ontology (GO) terms and Kyoto Encyclopedia of Genes and Genomes (KEGG) pathways were obtained from Molecular Signatures Database (MSigDB) [45].

### Consensus clustering analysis

To create clusters of gliomas based on members with similar intrinsic features of PDIs mRNA expression, samples were grouped into distinct subgroups by consensus clustering analysis with the R package “ConsensusClusterPlus” [46]. The subsampling parameter was 80% with 1000 times and k (the number of clusters) ranged from 2 to 10. The optimal number of clusters was determined according to cumulative distribution function plots and consensus matrices [47]. Differences in PDIs expression and clinical features of glioma between clusters were visualized by heat maps.

### Least absolute shrinkage and selection operator (LASSO) Cox regression

The LASSO Cox regression was used to construct a PDIs-based survival risk assessment model. Prognostic PDI family members were used to calculate the LASSO coefficients, according to the highest lambda value through Lasso method (lambda 1se) with 10-fold cross validation [48]. Subsequently, the risk score model of glioma was established based on LASSO coefficients. The association between risk score and PDIs expression and clinical characters was illustrated by heat maps and box plots.

### Survival analysis

Glioma patients were divided into high and low groups on the basis of median PDI signature or risk score. Survival curves were created by the Kaplan-Meier method with log-rank test, comparing the overall survival (OS), progression free interval (PFI), and disease specific survival (DSS) rates of patients in low and high group. Additionally, survival analyses were conducted between clusters of gliomas.

### Analysis of genetic alterations in gliomas

We further investigated genetic alterations in gliomas, including somatic mutations and somatic copy number alternations (SCNAs), based on samples obtained from TCGA database. Glioma samples were divided into four groups according to the values of PDI signature and risk scores of low and high groups. Thereafter, the top 20 genes with the highest mutation frequency in the 1^st^ (lowest) and 4^th^ (highest) groups were screened and presented. In addition, Genomic Identification of Significant Targets in Cancer 2·0 (GISTIC) was used to explore SCNAs between the two groups classified by median value of risk score, according to the gene copy number variation data and Human genome reference consortium h19 downloaded from TCGA and GISTIC 2·0 [49]. The threshold copy number at alteration peaks were derived from GISTIC analysis.

### Receiver operating characteristic (ROC)

ROC and the area under the curve (AUC) were used to compare the prediction performance of PDI signature, risk score, clustering and grade in several aspects, including 3-year OS, subtype (CL+ME, NE+PN), MGMT status (methylated, unmethylated), 1p19q codel status (codel, noncodel), and IDH status (wildtype, mutant).

### Cox regression analysis and nomogram construction

The univariate and multivariate Cox regression analyses were performed to identify prognostic factors with P value < 0·05 for designing a prognostic model. A nomogram was constructed using the “RMS” [50, 51] in R software to predict 3- and 5-year OS in accordance with the results of multivariate Cox regression analyses. The prediction accuracy of the nomogram for OS was evaluated by a calibration curve and AUC. Furthermore, the accuracy of the prognostic models was also validated in CGGA dataset.

### Cell culture

Human glioma cell lines (U251, T98G, A172, U343) were obtained from the Chinese Academy of Sciences (Shanghai, China). All cells were maintained in Dulbecco's modified Eagle's medium (DMEM) (Gibco, CA, USA) supplemented with 10% fetal bovine serum (FBS) (Gibco, CA, USA) at 37°C with 5% CO_2_.

### Quantitative real-time polymerase chain reaction (qRT-PCR) analysis

Total RNA was extracted from cells using RNAiso Plus reagent (Gibco, CA, USA) according to manufacturer’s instructions. The total RNA was reverse-transcribed into cDNA using the PrimeScript RT Master Mix (Takara, China) kit following the manufacturer’s recommendations. The qRT-PCR was conducted with the SYBR^®^ Premix DimerEraserTM (Takara, China) on the LightCycler^®^ 480 system (Roche Diagnostics, Basel, Switzerland). The following primer sequences were used: P4HB forward primer, 5′-GGTGCTGCGGAAAAGCAAC-3′ and reverse primer, 5′-ACCTGATCTCGGAACCTTCTG-3′; PDIA4 forward primer, 5′-GGCAGGCTGTAGACTACGAG-3′ and reverse primer, 5′-TTGGTCAACACAAGCGTGACT-3′; PDIA5 forward primer, 5′-AAAGGTCTCCTCGCTCATTGA-3′ and reverse primer, 5′-CACCAGTACATTATTCCGGGTTC-3′; TXNDC12 forward primer, 5′-TGGCAAGGTGCATCCTGAAAT-3′ and reverse primer, 5′-TGCTCGGCACTGACATAAAAA-3′. GAPDH was used as the internal reference gene for data normalization. The qRT-PCR reaction steps were as follows: denaturation at 95°C for 30 sec, followed by 45 cycles of 95°C for 5 sec and 60°C for 20 sec. The relative mRNA expression was analyzed using the 2^-ΔΔCt^ method.

### The human protein atlas and 3D protein structure

The immunohistochemical assay images of different PDI family members in the normal and high grade glioma tissues were downloaded from the Human Protein Atlas website (https://www.proteinatlas.org) [52, 53].

### Statistical analysis

All statistical analyses were conducted using R software (version 3·5·3). Differences between groups were assessed using the two-tailed Students’ t-test. Multiple groups were compared by one-way ANOVA test. The Schoenfeld residual plots was performed to assess the PH assumption. Univariate and multivariate Cox regression analyses were performed to determine the prognostic value of the risk score and clinical features. The partition around medoids (PAM) algorithm has been conducted in the consensus clustering analysis. The chi-squared test was performed, while analyzing clinical characteristics’ difference between cluster 1 and 2. Kaplan-Meier method with log-rank test was used to compare the OS, PFI and DSS rates of glioma patients (low-/high-PDI signature, low-/high-risk score, cluster ½). The correlation between two variables was analyzed with Spearman rank. *p* < 0·05 was considered to indicate statistical significance.

## Supplementary Material

Supplementary Figures

Supplementary Table 1

Supplementary Table 2
