# Supplementary material for: Protein disulfide isomerases are promising targets for predicting the survival and tumor progression in glioma patients
Source: Aging (Albany NY). 2020 Feb 5;12(3):2347–72. doi: 10.18632/aging.102748 (PMC7041756; doi:10.18632/aging.102748)
Supplement: Supplementary Figures [file aging-12-102748-s003..pdf]

SUPPLEMENTARY FIGURES

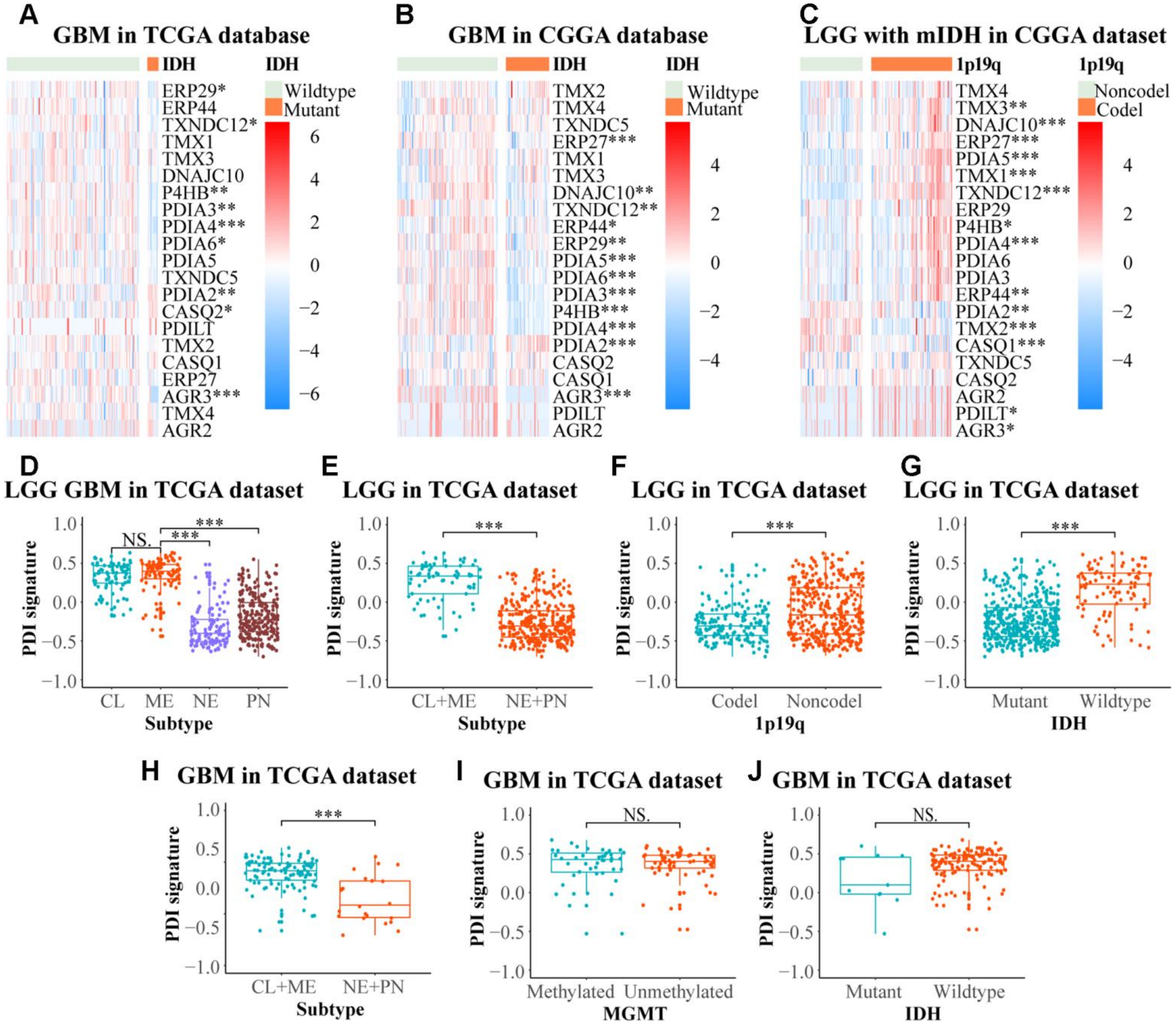

Supplementary Figure 1. (**A**, **B**) The mRNA expression patterns of PDIs in GBM with different status of IDH from TCGA and CGGA datasets. (**C**) The mRNA expression patterns of PDIs in LGG with different status of 1p19q from CGGA dataset. (**D**–**J**) In TCGA dataset, the distribution of PDI signature in gliomas was classified by subtype, 1p19q codel status, IDH status and MGMT promoter status. TCGA database as training set and CGGA database as the validation set. * P<0.05, ** P<0.01, ***P<0.001, NS. P>0.05.

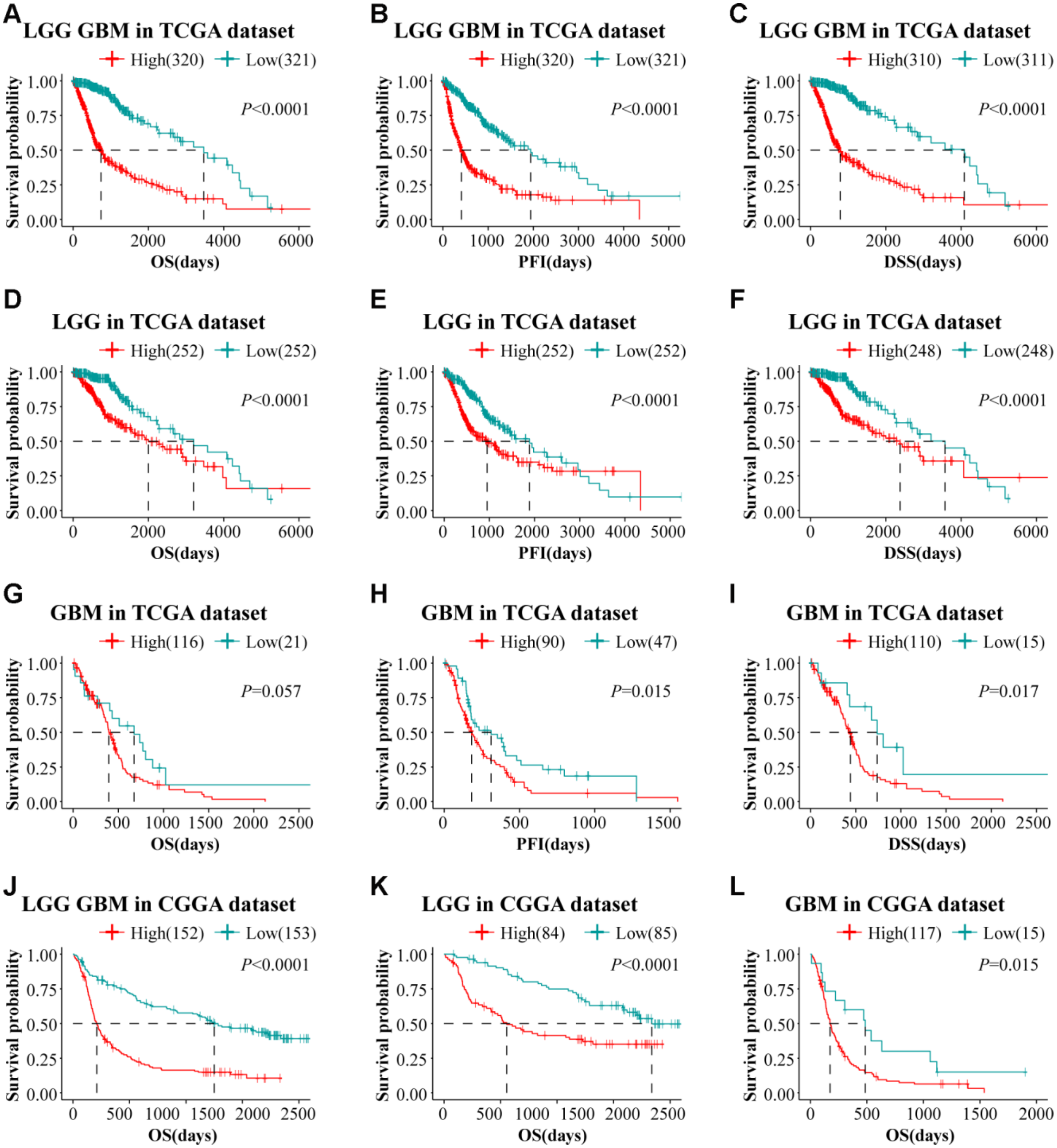

Supplementary Figure 2. The prognostic value of PDI signature in patients with gliomas. Kaplan-Meier survival curves for the OS, PFI and DSS, based on the value of PDI signature in LGGGBM (**A**–**C**), LGG (**D**–**F**), and GBM (**G**–**I**) samples downloaded from TCGA. (**J**–**L**) The survival curves of OS were constructed based on the CCGA datasets. TCGA database as training set and CGGA database as the validation set. CV, cut-off value used to divide patients into high and low groups.

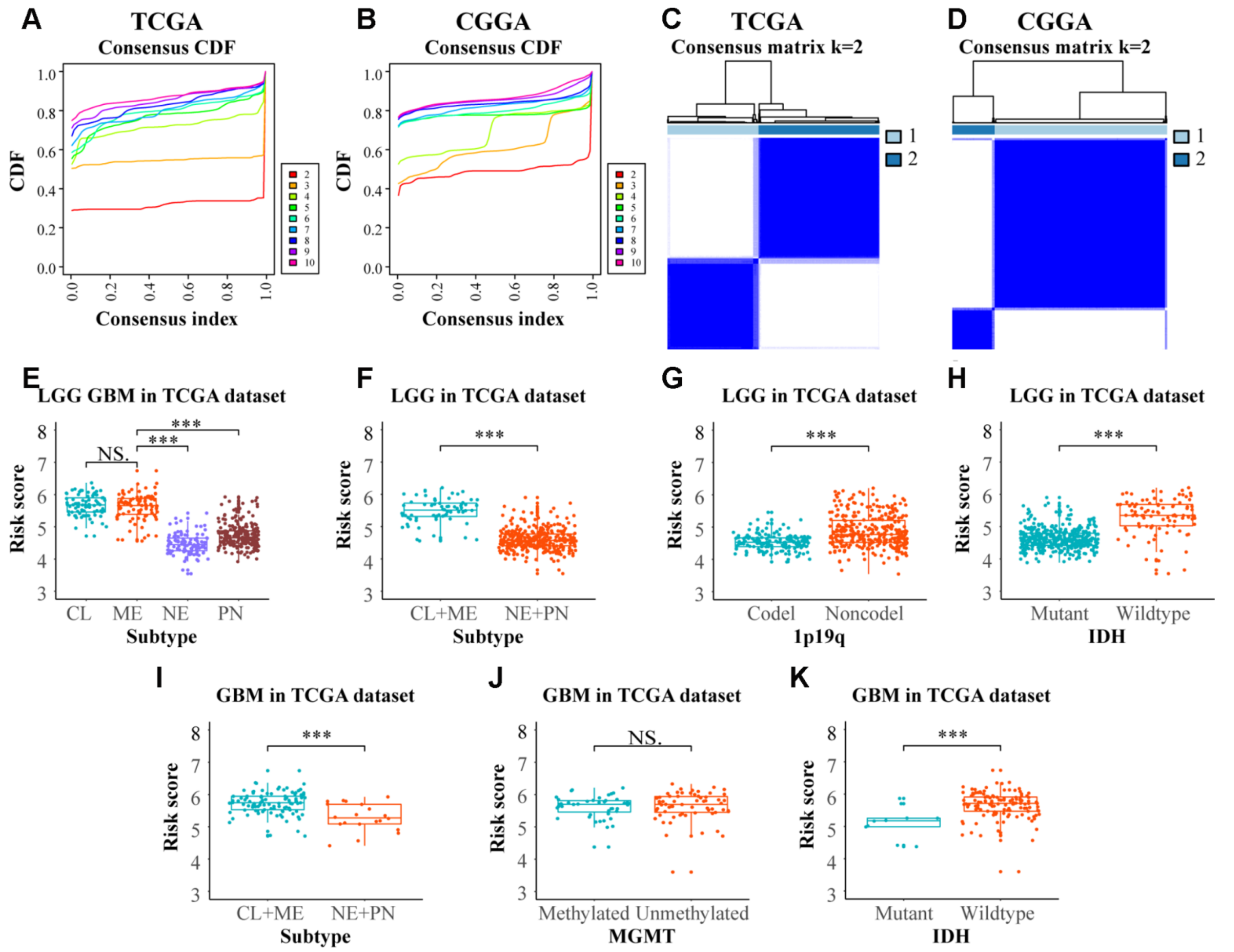

Supplementary Figure 3. (**A**, **B**) Clustering by the consensus clustering algorithm with k= 2 to 10. The cumulative distribution function (CDF) plot of the PDIs mRNA expression in gliomas from TCGA and CGGA. k=2 was defined as the optimal number. (**C**, **D**) Consensus matrix for 2 clusters. The dark blue rectangles show the samples assigned to the 2 clusters while the light blue lines represent the unassigned samples. Comparisons of risk score values between subgroups separated by clinicopathological characters. Differences in risk scores in LGGGBM (e), LGG (**F**, **H**) and GBM (**I**, **K**) classified by subtype, 1p19q codel status, IDH status and MGMT promoter status. ***P<0.001, NS P>0.05. TCGA database as training set and CGGA database as the validation set.

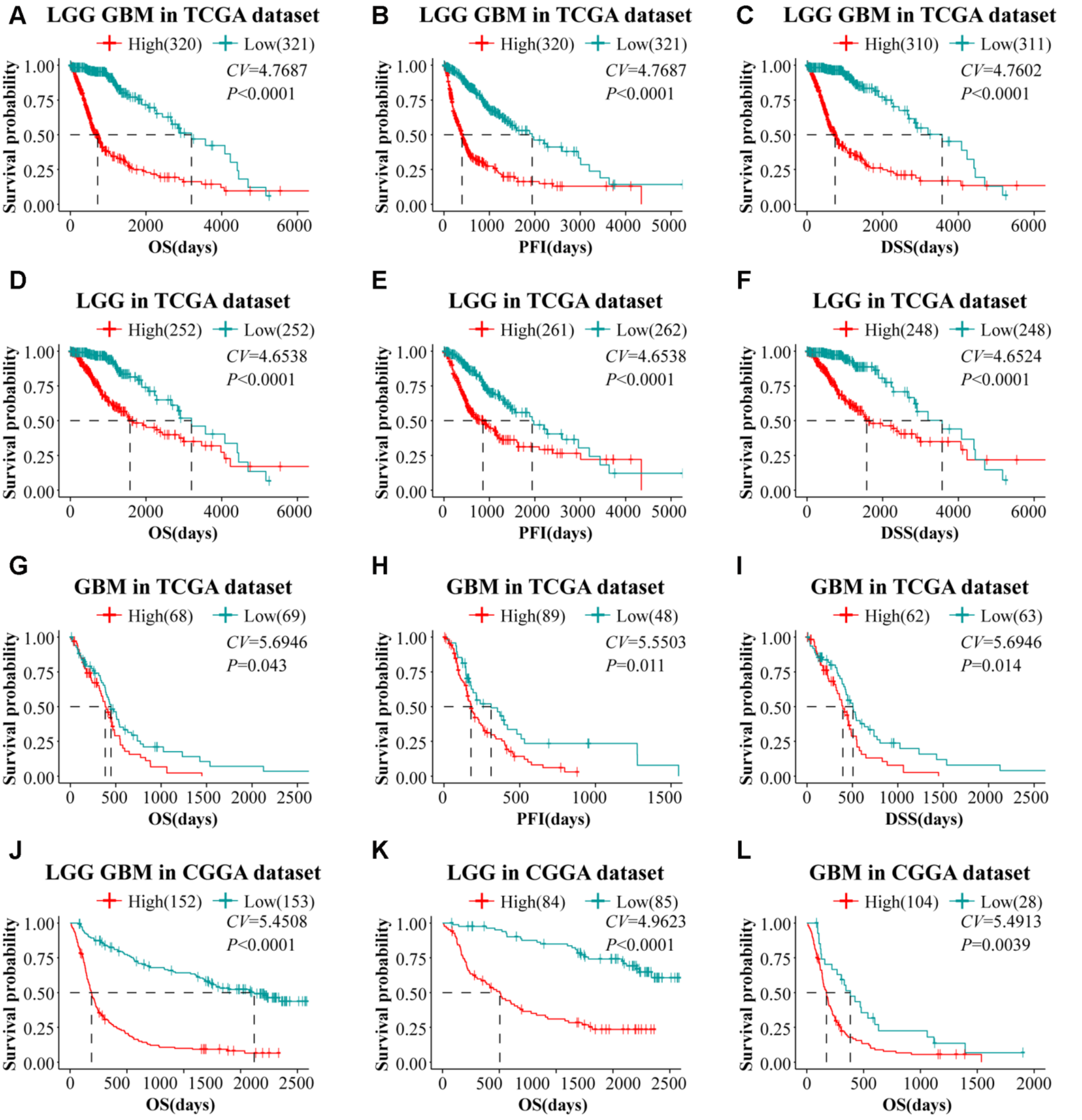

Supplementary Figure 4. The prognostic value of risk scores in patients with gliomas. Kaplan-Meier survival curves for the OS, PFI and DSS, according to the risk score values in LGGGBM (**A**–**C**), LGG (**D**–**F**), and GBM (**G**–**I**) samples downloaded from TCGA database. (**J**–**L**) The survival curves for the OS based on datasets from the CCGA database. TCGA database as training set and CGGA database as the validation set. CV, cut-off value used to divide patients into high and low groups.

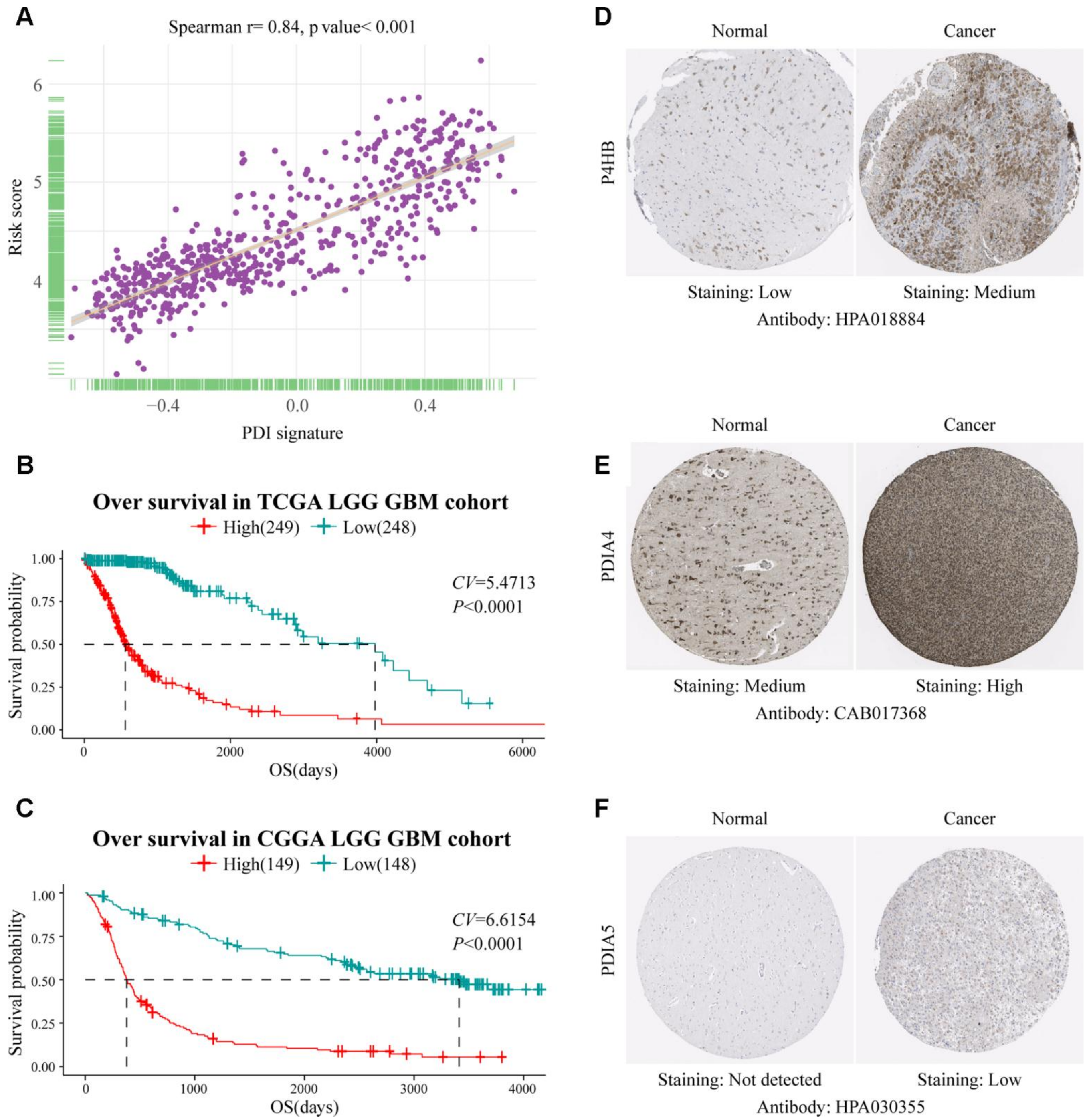

Supplementary Figure 5. (**A**) Correlation between PDI signature and risk score. (**B**–**C**);Comparison of OS between two groups divided by the median value of the prognostic model (Low vs High). Representative immunohistochemical staining images of P4HB (**D**), PDIA4 (**E**), and PDIA5 (**F**), in normal brain tissues and high grade glioma tissues, which were downloaded from the Human Protein Atlas website (www.proteinatlas.org). TCGA database as training set and CGGA database as the validation set. CV, cut-off value used to divide patients into high and low groups.
