# Supplementary material for: Protein disulfide isomerases are promising targets for predicting the survival and tumor progression in glioma patients
Source: Aging (Albany NY). 2020 Feb 5;12(3):2347–72. doi: 10.18632/aging.102748 (PMC7041756; doi:10.18632/aging.102748)
Supplement: Supplementary Table 1 [file aging-12-102748-s001..docx]

**Supplementary Table 2. Univariate and multivariate Cox regression analysis of factors affecting overall survival of patients.**

| Variables | | TCGA | | | | CGGA | | | | |
| --- | --- | --- | --- | --- | --- | --- | --- | --- | --- | --- |
|  |  | Univariate analysis | | Multivariate analysis | | Univariate analysis | | Multivariate analysis | | |
|  |  | P value | HR (95%CI) | P value | HR (95%CI) | P value | HR (95%CI) | | P value | HR (95%CI) |
| Risk score | OS | 8.19E-41 | 5.74 (4.45-7.42) | 0.0198 | 1.66 (1.08-2.55) | 3.83E-26 | 2.91 (2.39-3.55) | | 0.00723 | 1.56 (1.13-2.15) |
|  | PFI | 1.80E-37 | 4.51 (3.58-5.68) | 0.0019 | 1.83 (1.25-2.68) | \ | \ | | \ | \ |
|  | DSS | 2.78E-38 | 6.03 (4.59-7.91) | 0.0224 | 1.70 (1.08-2.68) | \ | \ | | \ | \ |
| WHO grade (III/II) | OS | 2.22E-8 | 3.47 (2.24-5.36) | 0.0021 | 2.08 (1.30-3.31) | 6.56E-09 | 3.63 (2.35-5.61) | | 1.78E-06 | 2.98 (1.90-4.66) |
|  | PFI | 0.0009 | 1.75 (1.26-2.43) | 0.7313 | 1.07 (0.74-1.54) | \ | \ | | \ | \ |
|  | DSS | 4.08E-08 | 3.70 (2.32-5.90) | 0.0032 | 2.13 (1.29-3.51) | \ | \ | | \ | \ |
| WHO grade (IV/II) | OS | 5.59E-36 | 17.67 (11.28-27.71) | 3.62E-6 | 3.86 (2.18-6.84) | 8.71E-25 | 8.29 (5.54-12.41) | | 3.64E-10 | 4.41 (2.77-7.01) |
|  | PFI | 9.13E-30 | 7.62 (5.37-10.83) | 0.0523 | 1.61 (1.00-2.60) | \ | \ | | \ | \ |
|  | DSS | 5.83E-32 | 18.13 (11.19-29.39) | 1.69E-05 | 3.82 (2.07-7.04) | \ | \ | | \ | \ |
| Gender (Male) | OS | 0.2595 | 1.18 (0.89-1.57) | 0.3548 | 1.15 (0.85-1.56) | 0.5709 | 0.92 (0.70-1.22) | | 0.6788 | 0.94 (0.70-1.26) |
|  | PFI | 0.6770 | 0.95 (0.73-1.22) | 0.7790 | 0.96 (0.74-1.25) | \ | \ | | \ | \ |
|  | DSS | 0.3270 | 1.17 (0.86-1.58) | 0.4888 | 1.12 (0.81-1.55) | \ | \ | | \ | \ |
| IDH (Wildtype) | OS | 6.44E-42 | 9.03 (6.57-12.41) | 0.0111 | 2.10 (1.18-3.71) | 1.46E-11 | 2.68 (2.01-3.57) | | 0.1604 | 0.75 (0.50-1.12) |
|  | PFI | 7.43E-39 | 6.81 (5.11-9.09) | 9.97E-5 | 2.70 (1.64-4.44) | \ | \ | | \ | \ |
|  | DSS | 9.33E-39 | 9.64 (6.85-13.56) | 0.0025 | 2.50 (1.38-4.54) | \ | \ | | \ | \ |
| 1p19q (Noncodel) | OS | 1.37E-10 | 4.73 (2.94-7.59) | 0.0392 | 1.84 (1.03-3.28) | 3.15E-12 | 5.98 (3.62-9.89) | | 2.35E-05 | 3.27 (1.89-5.66) |
|  | PFI | 1.25E-11 | 3.47 (2.42-4.98) | 0.0726 | 1.49 (0.96-2.31) | \ | \ | | \ | \ |
|  | DSS | 4.96E-10 | 5.14 (3.07-8.60) | 0.0457 | 1.89 (1.01-3.54) | \ | \ | | \ | \ |
| Age (≥45) | OS | 2.26E-17 | 4.23 (3.03-5.91) | 0.0006 | 2.12 (1.38-3.27) | 5.88E-6 | 1.91 (1.44-2.52) | | 0.5103 | 1.11 (0.81-1.54) |
|  | PFI | 5.36E-10 | 2.33 (1.78-3.04) | 0.5135 | 1.12 (0.79-1.59) | \ | \ | | \ | \ |
|  | DSS | 2.31E-15 | 4.19 (2.94-5.97) | 0.0012 | 2.12 (1.35-3.34) | \ | \ | | \ | \ |
| Subtype (NE+PN) | OS | 3.14E-34 | 0.15 (0.11-0.20) | 0.6728 | 1.11 (0.68-1.80) | 2.77E-17 | 0.28 (0.21-0.38) | | 0.5378 | 0.87 (0.55-1.36) |
|  | PFI | 8.22E-3 | 0.19 (0.15-0.25) | 0.9495 | 1.01 (0.64-1.61) | \ | \ | | \ | \ |
|  | DSS | 8.25E-32 | 0.14 (0.10-0.20) | 0.3427 | 1.30 (0.76-2.24) | \ | \ | | \ | \ |
