# Supplementary material for: Protein disulfide isomerases are promising targets for predicting the survival and tumor progression in glioma patients
Source: Aging (Albany NY). 2020 Feb 5;12(3):2347–72. doi: 10.18632/aging.102748 (PMC7041756; doi:10.18632/aging.102748)
Supplement: Supplementary Table 2 [file aging-12-102748-s002..pdf]

SUPPLEMENTARY TABLES

Please browse Full Text version to see the data of Supplementary Tables 1

Supplementary Table 1. Univariate and multivariate Cox regression analysis of factors affecting overall survival of patients.

Supplementary Table 2. Clinical information of glioma patients from TCGA and CGGA database.

Clinicopathological features	TCGA	CGGA
	n (n%)	n (n%)
Number of patients	641 (100%)	319 (100%)
Subtype		
Proneural	229 (35.73%)	101 (31.66%)
Classical	79 (12.32%)	71 (22.26%)
Neural	108 (16.85%)	81 (25.39%)
Mesenchymal	90 (14.04%)	66 (20.69%)
NA	135 (21.06%)	0 (0.00%)
MGMT		
Methylation	460 (71.76%)	\
Unmethylation	150 (23.40%)	\
NA	31 (4.84%)	\
1p19q		
Codeletion	165 (25.74%)	67 (21.00%)
Noncodeletion	472 (73.63%)	245 (76.80%)
NA	4 (0.63%)	7 (2.20%)
IDH		
Mutation	417 (65.05%)	174 (54.55%)
Wildtype	217 (33.85%)	145 (45.45%)
NA	7 (1.10%)	0 (0.00%)
Gender		
Male	371 (57.88%)	198 (62.07%)
Female	270 (42.12%)	121 (37.93%)
Age		
<45	315 (49.14%)	187 (58.62%)
≥45	326 (50.86%)	132 (41.38%)
Grade		
WHO grade II	244 (38.07%)	102 (31.97%)
WHO grade III	259 (40.41%)	77 (24.14%)
WHO grade IV	137 (21.37%)	136 (42.63%)
NA	1 (0.15%)	4 (1.26%)
Cancer type		
LGG	504 (78.63%)	179 (56.11%)
GBM	137 (21.37%)	136 (42.63%)
NA	0 (0.00%)	4 (1.26%)
